# Horizontal Pendular Nystagmus and Ataxia Secondary to Severe Hypomagnesemia

**DOI:** 10.5334/tohm.910

**Published:** 2024-07-22

**Authors:** Marcos Polanco, María Rivera, Leire Manrique, Carmen Lage, Jon Infante

**Affiliations:** 1 Neurology Service, University Hospital Marqués de Valdecilla-IDIVAL, Santander, Spain; 2 Universidad de Cantabria (UC), Spain; 3 Centro de Investigación Biomédica en Red Enfermedades Neurodegenerativas (CIBERNED), ISCIII, Spain

**Keywords:** Pendular nystagmus, hypomagnesemia, ataxia, cerebellum

## Abstract

**Background:**

Severe hypomagnesemia is an increasingly recognized cause of acute and reversible cerebellar ataxia, often accompanied by cerebellar oculomotor signs such as jerky horizontal or downbeat nystagmus and very rarely ocular flutter.

**Phenomenology Shown:**

This video illustrates horizontal pendular nystagmus in a patient with acute onset cerebellar ataxia associated with severe hypomagnesemia.

**Educational value:**

Acquired pendular nystagmus can be distinguished from macrosaccadic oscillations and ocular flutter in that the former is composed of two slow phases of equal velocity and the latter of two fast phases of saccadic type with or without intersaccadic interval, respectively. It is most commonly associated with demyelinating, toxic, metabolic, and genetic disorders, but has not been reported in association with severe hypomagnesemia.

Severe hypomagnesaemia in an increasingly recognized cause of acute and reversible cerebellar ataxia associated with certain ocular motor disorders and abnormal movements [1]. Here we describe a patient who presented with pendular nystagmus and vertigo secondary to severe hypomagnesemia.

A 64-year-old man was admitted for a 3-day history of abdominal pain, nausea, dizziness, diplopia, and oscillopsia. His medical history included hypertension, hypercholesterolemia, anticoagulated atrial fibrillation, and chronic anemia. He was also under chronic treatment with omeprazole. Three months earlier, he had a bowel perforation due to phlegmonous diverticulitis with complicated sepsis, associated renal failure, and mild concomitant hypomagnesemia, which resolved with oral treatment. In the week prior to the current symptoms, he had diarrhea and vomiting, attributed to viral gastroenteritis.

On physical examination, his speech, level of consciousness, and orientation were normal. Ocular examination revealed horizontal pendular nystagmus that changed to jerky downbeat nystagmus on lateral gaze (video). Initially, no gait or appendicular ataxia was observed, but these symptoms appeared in the following hours and in a severe way, preventing him from walking or standing up. A multimodal CT scan with perfusion and angiography showed no alterations. Laboratory tests revealed severe hypomagnesemia (<0.5 mg/dl (1.6-2-5)), mild hypokalemia (2.7 mEq/l (3.5–5.1)) and hypocalcemia (7.8 mg/dl (8.7–10.4)).

**Video Segment:** **1 at admission:** Oculomotor examination at admission showing horizontal pendular nystagmus at primary gaze position changing to downbeat nystagmus on bilateral eccentric gaze. **Segment 2 at follow-up:** Oculomotor examination 5 months after discharge showing mild torsional nystagmus on right lateral gaze.

The patient received a high-dose replacement therapy with intravenous magnesium (22.5 gr. in the first 24 h and 13.5 gr. along the following 15d). Hypokalemia and hypocalcemia were also corrected. Cranial gadolinium contrast MRI, carried out 13 days after the onset of symptoms, was unremarkable. The pendular nystagmus resolved within hours but a torsional nystagmus in the right lateral gaze persisted. Also, mild gait and bilateral appendicular ataxia persisted for 48 h. The patient was discharged with oral magnesium supplementation, and omeprazole was replaced by famotidine.

At follow-up five months later, the patient showed clinical improvement, with persistent mild diplopia in the right lateral gaze and minimal torsional nystagmus (video). Gait was stable, although cautious.

Hypomagnesemia is increasingly recognized as a reversible cause of acute cerebellar ataxia and ocular signs [2]. Eye signs reported to date include mainly jerk nystagmus, which is predominantly downbeat. We have not found descriptions of pendular nystagmus in this context. Acquired pendular nystagmus can be observed in several disorders among which multiple sclerosis is the most common [3].This type of nystagmus is characterized by the lack of a fast phase and must be differentiated from macrosaccadic oscillations or ocular flutter as the latter are saccadic jerk-type movements, with or without intersaccadic interval respectively, and therefore composed of two fast phases.
